# Demographic and Clinical Factors Associated With SARS-CoV-2 Anti-Nucleocapsid Antibody Response Among Previously Infected US Adults: The C4R Study

**DOI:** 10.1093/ofid/ofaf123

**Published:** 2025-03-20

**Authors:** Ryan T Demmer, Chaoqi Wu, John S Kim, Yifei Sun, Pallavi Balte, Mary Cushman, Rebekah Boyle, Russell P Tracy, Linda M Styer, Taison D Bell, Michaela R Anderson, Norrina B Allen, Pamela J Schreiner, Russell Bowler, David A Schwartz, Joyce S Lee, Vanessa Xanthakis, Jean M Rock, Rachel Bievenue, Amber Pirzada, Margaret Doyle, Elizabeth A Regan, Barry J Make, Alka M Kanaya, Namratha R Kandula, Sally E Wenzel, Josef Coresh, Carmen R Isasi, Laura M Raffield, Mitchell S V Elkind, Virginia J Howard, Victor E Ortega, Prescott Woodruff, Shelley A Cole, Joel M Henderson, Nicholas J Mantis, Elizabeth C Oelsner

**Affiliations:** Division of Epidemiology, Department of Quantitative Health Sciences, College of Medicine and Science, Mayo Clinic, Rochester, Minnesota, USA; Department of Epidemiology, Columbia University Mailman School of Public Health, New York, New York, USA; Division of Epidemiology and Community Health, School of Public Health, University of Minnesota, Minneapolis, Minnesota, USA; Department of Medicine, Columbia University Vagelos College of Physicians and Surgeons, New York, New York, USA; Department of Medicine, Columbia University Vagelos College of Physicians and Surgeons, New York, New York, USA; Department of Medicine, University of Virginia School of Medicine, Charlottesville, Virginia, USA; Department of Biostatistics, Columbia University Mailman School of Public Health, New York, New York, USA; Department of Medicine, Columbia University Vagelos College of Physicians and Surgeons, New York, New York, USA; Department of Medicine, Larner College of Medicine at the University of Vermont, Burlington, Vermont, USA; Department of Pathology and Laboratory Medicine, Larner College of Medicine at the University of Vermont, Burlington, Vermont, USA; Department of Pathology and Laboratory Medicine, Larner College of Medicine at the University of Vermont, Burlington, Vermont, USA; Department of Pathology and Laboratory Medicine, Larner College of Medicine at the University of Vermont, Burlington, Vermont, USA; Division of Infectious Diseases, Wadsworth Center, New York State Department of Health, Albany, New York, USA; Department of Medicine, University of Virginia School of Medicine, Charlottesville, Virginia, USA; Department of Medicine, University of Pennsylvania, Philadelphia, Pennsylvania, USA; Department of Preventive Medicine, Northwestern University Feinberg School of Medicine, Chicago, Illinois, USA; Division of Epidemiology and Community Health, School of Public Health, University of Minnesota, Minneapolis, Minnesota, USA; Division of Pulmonary, Critical Care and Sleep Medicine, National Jewish Health, Denver, Colorado, USA; Department of Medicine, University of Colorado School of Medicine, Aurora, Colorado, USA; Department of Medicine, University of Colorado School of Medicine, Aurora, Colorado, USA; Department of Medicine, Boston University Chobanian and Avedisian School of Medicine, Boston, Massachusetts, USA; Department of Medicine, College of Medicine and Deparmtne of Biostatistics, School of Public Health, Boston University, Boston, Massachusetts, USA; Division of Infectious Diseases, Wadsworth Center, New York State Department of Health, Albany, New York, USA; Division of Infectious Diseases, Wadsworth Center, New York State Department of Health, Albany, New York, USA; Institute for Minority Health Research, University of Illinois, College of Medicine, Chicago, Illinois, USA; Department of Medicine, Larner College of Medicine at the University of Vermont, Burlington, Vermont, USA; Division of Rheumatology, National Jewish Health, Denver, Colorado, USA; Division of Pulmonary, Critical Care and Sleep Medicine, National Jewish Health, Denver, Colorado, USA; Division of General Internal Medicine, University of California San Francisco, San Francisco, California, USA; Department of Preventive Medicine, Northwestern University Feinberg School of Medicine, Chicago, Illinois, USA; Department of Medicine, Department of Immunology, and Department of Environmental Medicine and Occupational Health, University of Pittsburgh School of Medicine, School of Public Health, Pittsburgh, Pennsylvania, USA; Department of Medicine, Johns Hopkins University School of Medicine, Baltimore, Maryland, USA; Department of Epidemiology and Welch Center for Prevention, Epidemiology, and Clinical Research, Johns Hopkins Bloomberg School of Public Health, Baltimore, Maryland, USA; Department of Epidemiology and Population Health, Albert Einstein College of Medicine, Bronx, New York, USA; Department of Genetics, University of North Carolina, Chapel Hill, North Carolina, USA; Department of Epidemiology, Columbia University Mailman School of Public Health, New York, New York, USA; Department of Neurology, Columbia University Vagelos College of Physicians and Surgeons, New York, New York, USA; Department of Epidemiology, School of Public Health, University of Alabama at Birmingham, Birmingham, Alabama, USA; Division of Respiratory Medicine, Mayo Clinic, Scottsdale, Arizona, USA; Division of Pulmonary and Critical Care Medicine, University of California San Francisco, San Francisco, California, USA; Population Health Program, Texas Biomedical Research Institute, San Antonio, Texas, USA; Department of Pathology and Laboratory Medicine, Boston University Chobanian & Avedisian School of Medicine and Boston Medical Center, Boston, Massachusetts, USA; Division of Infectious Diseases, Wadsworth Center, New York State Department of Health, Albany, New York, USA; Department of Medicine, Columbia University Vagelos College of Physicians and Surgeons, New York, New York, USA

**Keywords:** adaptive immune response, anti-nucleocapsid antibodies, COVID-19, SARS-CoV-2 infection, serological studies

## Abstract

Despite the availability of effective vaccines and a recent decrease in annual deaths, COVID-19 remains a leading cause of death. Serological studies provide insights into host immunobiology of adaptive immune response to infection, which holds promise for identifying high-risk individuals for adverse COVID-19 outcomes. We investigated correlates of anti-nucleocapsid antibody responses following SARS-CoV-2 infection in a US population-based meta-cohort of adults participating in longstanding National Institutes of Health–funded cohort studies. Anti-nucleocapsid antibodies were measured from dried blood spots collected between February 2021 and February 2023. Among 1419 Collaborative Cohort of Cohorts for COVID-19 Research participants with prior SARS-CoV-2 infection, the mean age (standard deviation) was 65.8 (12.1), 61% were women, and 42.8% self-reported membership in a race/ethnicity minority group. The proportion of participants reactive to nucleocapsid peaked at 69% by 4 months after infection and waned to only 44% ≥12 months after infection. Higher anti-nucleocapsid antibody response was associated with older age, Hispanic or American Indian Alaskan Native (vs White) race/ethnicity, lower income, lower education, former smoking, and higher anti-spike antibody levels. Asian race (vs White) and vaccination (even after infection) were associated with lower nucleocapsid reactivity. Neither vaccine manufacturer nor common cardiometabolic comorbidities were not associated with anti-nucleocapsid response. These findings inform the underlying immunobiology of adaptive immune response to infection, as well as the potential utility of anti-nucleocapsid antibody response for clinical practice and COVID-19 serosurveillance.

The persistence of SARS-CoV-2 variants poses an ongoing challenge to public health even as the virus transitions to endemic status. COVID-19 remains a top 10 leading cause of death as of 2023, and post–COVID-19 conditions affect a considerable proportion of survivors [1]. Identifying individuals and populations at high risk for adverse COVID-19 outcomes is a high priority.

Serological studies provide insights into host immunobiology of adaptive immune response to infection. This knowledge holds promise for identifying high-risk individuals for adverse COVID-19 outcomes, including acute and postacute conditions, and the development of prevention and treatment strategies. Although prior work has examined postinfection kinetics and correlates of the anti-spike (S) antibody response [2–6], correlates of the anti-nucleocapsid (N) antibody response—which is only generated by natural infection, not by vaccination—are relatively understudied and have important limitations [7–17]. Milder acute infection severity and history of COVID-19 vaccination have been linked to lower anti-N responses in some studies, yet results have been mixed. Prior studies also had one of several of the following notable limitations including small sample sizes, limited representation of ages >65 years, lack of precise information on timing of infection, inability to account for potentially important risk factors (eg, smoking status, adiposity, clinical conditions), no inclusion of vaccinated individuals, lack of racial diversity, and study completion before the emergence of the less virulent Omicron variant.

In addition, anti-N serum immunoglobulin G (IgG) antibodies are used to classify history of SARS-CoV-2 infection in clinical practice, surveillance, and research. Differential anti-N antibody response may lead to differential misclassification and inclusion in studies of post-acute sequelae of SARS-CoV-2 infection. Hence, understanding the correlates of the anti-N response may inform strategies to reduce misclassification and selection bias in clinical and research settings.

We investigated the correlates of the anti-N antibody response following SARS-CoV-2 infection in a large, diverse, US population-based meta-cohort of adults. We assessed the dynamics of the antibody response over time since infection. Then, accounting for temporal trends, we examined the association of anti-N response with sociodemographics, prepandemic health and lifestyle factors, acute illness severity, and COVID-19 vaccination history.

## METHODS

### Participants

The Collaborative Cohort of Cohorts for COVID-19 Research (C4R) is performing standardized assessments of COVID-19 in participants in 14 longstanding National Institutes of Health (NIH)–funded prospective cohort studies (see Supplementary Methods) [18] to study the impact of the COVID-19 pandemic on US adults. Cohort participants who were alive on 1 March 2020, and had not withdrawn consent for cohort participation, were considered eligible for C4R enrollment.

Inclusion criteria for the present analysis were prior SARS-CoV-2 infection, assessed via self-report and/or review of hospital discharge records, prior to submission of a valid dried blood spot (DBS) or a venous serum sample for serology assay (Supplementary Figure 1).

### Patient Consent Statement

Columbia University served as the Data Coordination and Harmonization Center for C4R (Columbia University institutional review board, AAAT3035). Institutional review board approval was obtained from all study sites [18]. Informed consent was obtained from each study participant (Supplementary Materials).

### Serosurvey

C4R serological assays were performed on DBS, as previously described [18, 19]. The DBS was created by depositing drops of whole blood from a finger prick or blood collection tube onto a bar-coded Whatmann 903 filter paper card. Participants completed the DBS at home or at an in-person examination. DBS samples were shipped via US mail to the University of Vermont, and per the Centers for Disease Control and Prevention–recommended protocol samples were stored at −80 °C within 2 weeks of collection to loss of sample quality [20]. DBS samples were collected between February 2021 and February 2023.

Serological assays were performed on DBS eluates by the Wadsworth Center, New York State Department of Health (Albany, NY, USA), using validated methods [21]. The assays detect IgG for 4 SARS-CoV-2 spike antigens (receptor binding domain, S1 protein, full length spike, and spike trimer), which may be induced by natural infection or COVID-19 vaccines, and 3 different varieties of N protein, which is induced by natural infection only. Assays covalently coupled SARS-CoV-2 spike and N antigens [21] to Magplex-C microspheres (Luminex Corp., Austin, TX, USA) with different bead regions coupled to each antigen. Beads and DBS eluates were added to a 384-well plate and incubated for 30 minutes at 37 °C while shaking. After washing on a magnetic plate washer, phycoerythrin-labelled anti-IgG antibody was added to the plate and incubated for 30 minutes at 37 °C while shaking. The plate was washed and beads were resuspended with buffer. A FlexMap 3D instrument (Luminex Corp.) analyzed median fluorescence intensity (MFI). One lot of beads and 2 lots of anti-IgG antibody were used over the period of analysis. See Supplementary supplemental information for reagent details.

Microsphere immunoassays are multiplex assays in which each bead set can be analyzed independently from the others. In this study, we have analyzed the N protein (Sino Biological, Wayne, PA, USA).

We classified antibody response as reactive or nonreactive based on the mean and standard deviation (SD) of anti-N MFI values in uninfected (prepandemic) DBS samples [19]. To enhance the specificity of our assay, for each bead set, the reactivity threshold was calculated as the mean +6 SDs of the uninfected (prepandemic) MFI.

### Infection and Vaccination History

C4R collected information on SARS-CoV-2 infection and vaccination status via 2 waves of questionnaires conducted from April 2020 through February 2023 [22]; the current report includes data from questionnaires collected through February 2023. Questionnaires were administered by telephone interview, electronic survey, in-person examination, and/or mailed pamphlet. Participants were asked about history of SARS-CoV-2 infection, SARS-CoV-2 testing, COVID-19 hospitalization, COVID-19 vaccination status, date of first vaccine administration, number of vaccines received, and vaccine manufacturer (all vaccines included presently target only the spike antigen). Infections were dichotomized according to whether they were or were not associated with hospitalization. Confirmed history of SARS-CoV-2 infection was classified based on self-report of a positive SARS-CoV-2 test (antigen or polymerase chain reaction) or adjudication of medical records for a COVID-19 hospitalization; self-reported infections not meeting either of these criteria were classified as probable and the sample removed.

### Pre-pandemic Measures

C4R cohorts have performed longitudinal data collection on participants for up to age 51 years. C4R harmonized these data across cohorts, as previously described [18]. Age, sex, and educational attainment were self-reported. Race and ethnicity, which were self-reported and categorized according to the 2000 Census methods [23, 24], were included in this study to address specific knowledge gaps about disparities in COVID-19 outcomes among members of historically marginalized or underserved populations. Time-varying risk factors were defined using the most recent data collected by each cohort. Smoking status was self-reported as never, former, or current. Height, weight, blood pressure, fasting lipids, and blood glucose were measured using standardized protocols [18]. Body mass index was calculated and classified into standard Centers for Disease Control and Prevention–defined categories. Hypertension was defined as a systolic blood pressure ≥140 mm Hg, diastolic blood pressure ≥90 mm Hg, or antihypertensive medication use. Diabetes was defined as fasting blood glucose ≥126 mg/dL or use of diabetes medications. Cardiovascular disease, asthma, and chronic obstructive pulmonary disease were identified via self-report or by the ascertainment of relevant clinical events, confirmed by medical record review, over cohort follow-up.

### Statistical Analysis

Participant characteristics were described as mean ± SD for the full sample and mean ± standard error (SE) among subgroups for continuous variables; categorical variables are reported as n (%). Continuous variables with evidence of nonnormality were natural log-transformed prior to analysis.

Poisson models (with robust error variance) regressed the probability of N reactivity on candidate risk factors, time since infection (defined as days from infection to serosurvey), and laboratory batch. Secondary analyses regressed continuous natural log-transformed anti-nucleocapsid IgG levels on the aforementioned variables using generalized linear models. For linear regressions estimating continuous anti-nucleocapsid MFI levels, we exponentiated the β-coefficient and presented the results as mean percent difference in anti-nucleocapsid level in the comparison versus reference group.

Subgroup analyses were conducted to evaluate associations of severity of clinical infection with aspects of vaccination history, including timing of vaccination (before vs after infection) and manufacturer.

Multiple imputation by chained equations imputed missing covariates (Supplementary Table 1). Ten imputed datasets were created and results were combined using Rubin's rule [25]. Characteristics of complete cases were similar to the imputed (primary) dataset (Supplementary Table 2).

Our sample size proves >90% to detect a risk ratio for IgG reactivity >1.25% and 80% power to detect risk ratios >1.16 enabling reliable detection of small associations.

All analyses were conducted in R (R Statistical Foundation, Vienna, Austria) version 4.1 [18]. R packages used for analysis include: tidyverse (v1.3.1), arsenal (v3.6.3), mice (v3.17.0), ggplot2 (v3.3.5), and lmtest (v0.9.39). Two-sided *P*-values < .05 were considered statistically significant.

## RESULTS

### Participant Characteristics

Among 1419 C4R participants with evidence of prior SARS-CoV-2 infection, the mean age (SD) was 65.8 (12.1) years, 61% were women, and 57% self-reported race/ethnicity as White non-Hispanic (Table 1). Chronic disease prevalence and/or cardiometabolic risk factor levels were high: >80% of this sample was overweight or obese, 57% had hypertension, and 24% had diabetes. Participant characteristics according to N reactivity or MFI levels are summarized in Table 1 and Supplementary Table 3.

**Table 1.** Baseline Characteristics According to IgG Nucleocapsid Antibody Reactivity Status Among n = 1419 Participants in the C4R Study

Characteristic	Overall	Anti-N Reactive (n = 723)	Anti-N Not Reactive (n = 696)
No. of participants	1419	723	696
Anti-S1 antibody MFI (log-transformed)	9.0 (1.3)	9.3 (1.0)	8.7 (1.5)
Anti-N antibody MFI (log-transformed)	7.3 (1.5)	8.4 (0.8)	6.1 (1.0)
% S1-reactive	1393 (98.2%)	721 (99.7%)	672 (96.6%)
Age			
<50 y	132 (9.4%)	66 (9.2%)	66 (9.5%)
50–64 y	553 (39.2%)	268 (37.5%)	285 (40.9%)
65–79 y	588 (41.7%)	286 (40.1%)	302 (43.4%)
≥80 y	137 (9.7%)	94 (13.2%)	43 (6.2%)
Female	867 (61.1%)	439 (60.7%)	428 (61.6%)
Income			
<50,000	294 (54.9%)	171 (59.2%)	123 (49.8%)
50,000–100,000	141 (26.3%)	79 (27.3%)	62 (25.1%)
>100,000	101 (18.8%)	39 (13.5%)	62 (25.1%)
Self-reported race or ethnicity			
Non-Hispanic White	811 (57.2%)	396 (54.8%)	415 (59.7%)
Black	287 (20.2%)	149 (20.6%)	138 (19.9%)
Hispanic/Latino	66 (4.7%)	28 (3.9%)	38 (5.5%)
Asian	21 (1.5%)	13 (1.8%)	8 (1.2%)
American Indian	233 (16.4%)	137 (18.9%)	96 (13.8%)
Education attainment			
Less than high school	106 (7.8%)	60 (8.7%)	46 (6.9%)
High school	359 (26.4%)	200 (29.0%)	159 (23.7%)
Some college	354 (26.0%)	183 (26.5%)	171 (25.5%)
College	542 (39.8%)	247 (35.8%)	295 (44.0%)
Study cohort			
ARIC	70 (4.9%)	50 (6.9%)	20 (2.9%)
CARDIA	170 (12.0%)	85 (11.8%)	85 (12.2%)
COPDGene	187 (13.2%)	92 (12.7%)	95 (13.6%)
FHS	140 (9.9%)	60 (8.3%)	80 (11.5%)
JHS	26 (1.8%)	13 (1.8%)	13 (1.9%)
MASALA	12 (0.8%)	7 (1.0%)	5 (0.7%)
MESA	130 (9.2%)	58 (8.0%)	72 (10.3%)
PrePF	30 (2.1%)	14 (1.9%)	16 (2.3%)
REGARDS	345 (24.3%)	163 (22.5%)	182 (26.1%)
SARP	23 (1.6%)	16 (2.2%)	7 (1.0%)
SHS	231 (16.3%)	136 (18.8%)	95 (13.6%)
SPIROMICS	55 (3.9%)	29 (4.0%)	26 (3.7%)
Smoking status			
Never	633 (44.7%)	297 (41.2%)	336 (48.3%)
Former	576 (40.7%)	327 (45.4%)	249 (35.8%)
Current	207 (14.6%)	97 (13.5%)	110 (15.8%)
Body mass index, kg/m^2^			
<25 kg/m^2^	287 (20.6%)	144 (20.3%)	143 (21.0%)
25–29.9 kg/m^2^	470 (33.8%)	230 (32.4%)	240 (35.3%)
30–34.9 kg/m^2^	330 (23.7%)	168 (23.7%)	162 (23.8%)
>35 kg/m^2^	303 (21.8%)	168 (23.7%)	135 (19.9%)
Hypertension	796 (56.5%)	435 (60.5%)	361 (52.3%)
Diabetes	334 (23.8%)	185 (25.9%)	149 (21.7%)
Cardiovascular disease	146 (10.8%)	81 (11.7%)	65 (9.8%)
Chronic obstructive pulmonary disease	118 (11.4%)	69 (12.8%)	49 (9.8%)
COVID-19 infection severity			
Not hospitalized	1102 (77.8%)	553 (76.5%)	549 (79.1%)
Noncritical hospitalization	248 (17.5%)	126 (17.4%)	122 (17.6%)
Critical hospitalization	67 (4.7%)	44 (6.1%)	23 (3.3%)
Vaccine status			
Not vaccinated	234 (17.6%)	149 (22.2%)	85 (12.9%)
Vaccinated after infection	891 (66.9%)	422 (62.9%)	469 (71.0%)
Vaccinated before infection	207 (15.5%)	100 (14.9%)	107 (16.2%)
Vaccinated before infection	328.9 (177.6)	311.2 (177.3)	347.4 (176.2)
Vaccine manufacturer^a^			
Moderna	421 (42.3%)	195 (43.0%)	226 (43.5%)
Pfizer	488 (49.0%)	219 (48.3%)	269 (51.7%)
J&J	45 (4.5%)	23 (5.1%)	22 (4.2%)
Other	19 (1.9%)	16 (3.5%)	3 (0.58%)

IgG, immunoglobulin G; MFI, median fluorescence intensity; N, nucleocapsid.

^a^Vaccine manufacturer information is based on a denominator of n = 995 vaccinated participants with complete manufacturer information.

### Nucleocapsid Reactivity and Time Since Infection

Overall, 51.0% of participants were classified as N-reactive. N-reactivity was highest in the 1- to 6-month postinfection period, plateaued, and declined thereafter (Table 2 and Supplementary Table 4). Among participants with self-reported infection 12 or more months before the serosurvey, only 44.3% were classified as N-reactive. The date range of reported infections was January 2020—August 2022, which covers periods when either the Delta or Omicron variants were dominant in the United States.

**Table 2.** Proportion of Participants With IgG Nucleocapsid Reactivity Derived From Multivariable Adjusted Poisson Regression Models Among n = 1419 Participants in the C4R Study

Group	0–30 D (n = 15)	31–89 D (n = 92)	90–119 D (n = 62)	120–180 D (n = 132)	181–365 D (n = 608)	>365 D (n = 510)
Overall	33.33%	56.52%	69.35%	62.12%	51.81%	44.31%
Vaccine status						
Unvaccinated	50.00%	90.48%	84.21%	85.07%	56.21%	53.67%
Vaccinated after infection	n/a	40.00%	69.34%	52.22%	50.98%	43.32%
Vaccinated before infection	30.77%	46.97%	63.71%	51.87%	49.57%	37.69%
Age group						
<50 y	50.00%	86.30%	71.43%	50.62%	31.10%	58.88%
50–64 y	66.67%	50.43%	75.00%	66.98%	47.07%	42.47%
65–79 y	12.50%	45.83%	46.67%	61.08%	53.66%	40.43%
≥80 y	50.00%	71.43%	87.50%	59.82%	73.95%	58.90%
Sex						
Female	33.33%	61.82%	70.27%	63.33%	51.54%	42.13%
Male	33.33%	48.65%	68.00%	59.52%	52.19%	48.08%
Self-reported race or ethnicity						
Non-Hispanic White	20.00%	53.45%	69.77%	62.89%	50.77%	34.68%
Black	33.33%	54.55%	61.54%	70.59%	51.00%	49.44%
Hispanic/Latino	n/a	66.67%	n/a	37.50%	50.00%	35.48%
Asian	n/a	0.00%	0.00%	n/a	83.33%	42.86%
American Indian	100.00%	68.42%	100.00%	60.00%	53.66%	58.26%
Education attainment						
Less than high school	54.55%	61.54%	9.09%	71.43%	53.83%	58.20%
High school	69.70%	63.38%	77.05%	72.88%	55.56%	48.15%
Some college	31.82%	52.04%	69.01%	69.01%	52.11%	46.47%
College or beyond	1.96%	53.17%	66.90%	51.67%	48.83%	36.84%
Smoking status						
Never	42.86%	60.00%	71.33%	65.22%	46.22%	39.49%
Former	28.57%	56.41%	73.18%	61.19%	60.92%	48.63%
Current	0.00%	50.00%	50.00%	57.89%	42.42%	47.17%
Body mass index, kg/m^2^						
<25 kg/m^2^	9.09%	56.11%	68.57%	65.99%	49.78%	43.26%
25–29.9 kg/m^2^	75.00%	48.67%	54.29%	58.87%	48.99%	44.32%
30–34.9 kg/m^2^	24.49%	50.35%	79.88%	60.13%	50.34%	46.12%
>35 kg/m^2^	7.69%	72.09%	88.68%	65.78%	60.86%	43.35%
Hypertension						
No	50.00%	56.10%	55.17%	55.17%	46.33%	40.39%
Yes	22.22%	56.86%	81.82%	67.57%	56.30%	47.10%
Diabetes						
No	39.66%	61.54%	69.17%	61.54%	48.20%	43.16%
Yes	11.76%	28.57%	70.30%	64.29%	62.57%	47.74%
Cardiovascular disease						
No	26.87%	57.09%	70.07%	62.08%	50.81%	44.11%
Yes	87.50%	52.29%	61.54%	62.37%	60.00%	46.17%
COPD						
No	33.56%	55.57%	70.84%	60.23%	50.55%	43.48%
Yes	n/a	61.87%	0.00%	83.33%	59.71%	49.92%
COVID-19 infection severity						
Not hospitalized	35.71%	60.46%	67.31%	60.00%	48.87%	44.44%
Noncritical hospitalization	0.00%	15.87%	77.78%	70.59%	61.39%	38.60%
Critical hospitalization	n/a	33.33%	100.00%	80.00%	72.73%	61.11%

C4R, Collaborative Cohort of Cohorts for COVID-19 Research; COPD, chronic obstructive pulmonary disease; IgG, immunoglobulin G; n/a, not available.

### Correlates of Nucleocapsid Reactivity

**Figure 1.**
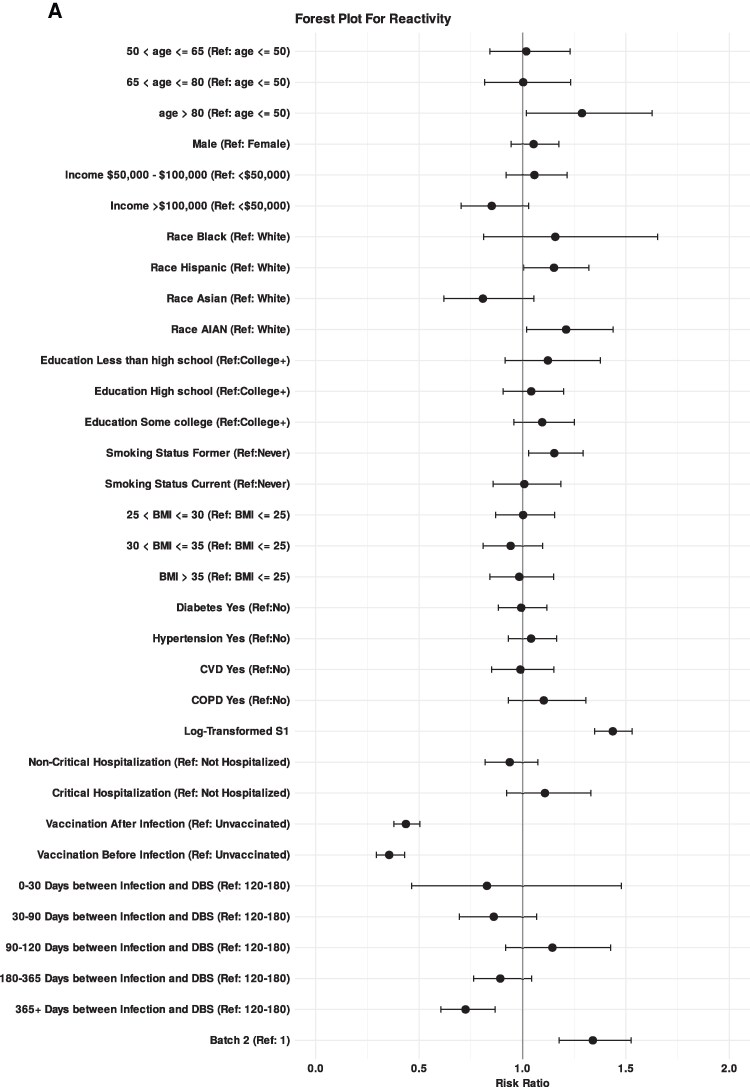

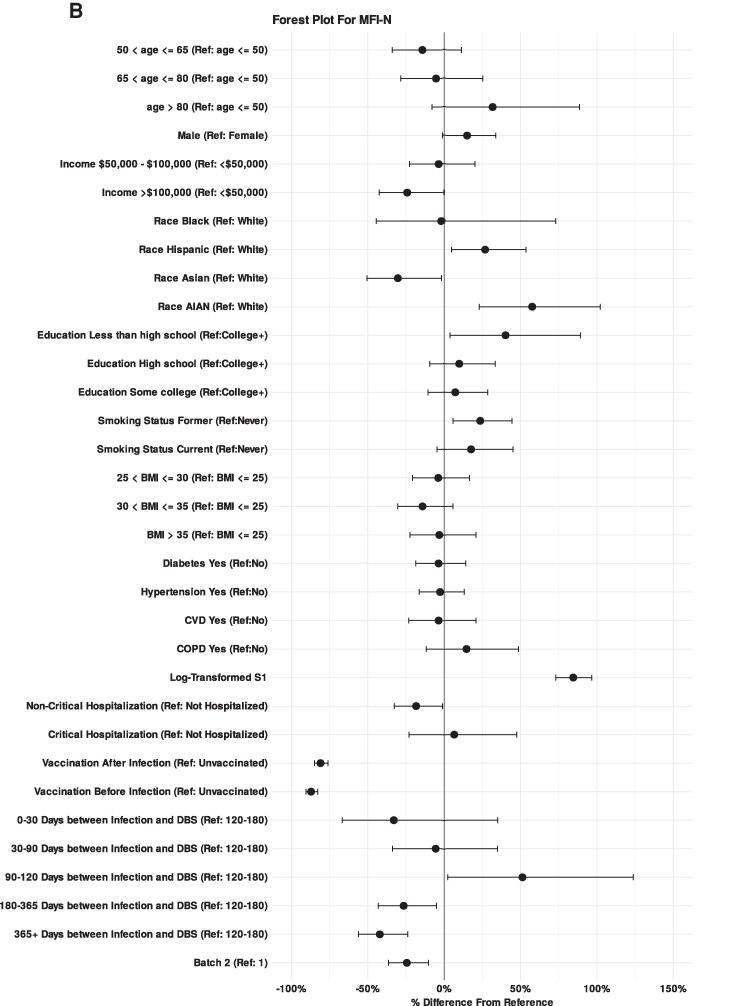
Forest plot showing correlates of anti-nucleocapsid antibody reactivity (*A*) and mean florescence unit (*B*) among n = 1419 Collaborative Cohort of Cohorts for COVID-19 Research (C4R) participants. n/a refers to categories without any participants and 0% indicated that although there were participants in the category, none of them were anti-nucleocapsid reactive. A total of 98% of participants in the analysis reported receiving an mRNA vaccine while the remaining 2% reported “other.” Plotted points represent the risk ratio for respective covariates and error bars indicate 95% confidence intervals.

After multivariable adjustment, increased probability of N-reactivity was associated with older age (≥80 vs <50 years), American Indian or Hispanic/Latino race, former smoker, higher anti-S1 MFI, and being unvaccinated at time of serosurvey; Asian race and >1 year between infection and DBS were associated with an decreased probability of N-reactivity (Table 3, Figure 1*A*); multivariable adjustment had minimal influence on these crude association results. Results were generally consistent in multivariable linear regressions assessing correlates of anti-N MFI levels with the following exceptions: (1) results for older age were not statistically significant, (2) less than high school eduction was associated with higher MFI levels, (3) MFI levels were lower among individuals with noncritical hospitalization (vs nonhospitalized), and (4) MFI levels appeared to wane by month 4 following infection (Figure 1*B*, Supplementary Table 5).

**Table 3.** Correlates of IgG Nucleocapsid Reactivity After COVID-19 Infection, From Multivariable Adjusted Poisson Regression Among n = 1419 Participants in the C4R Study

Clinical Risk Factor	Unadjusted Risk Ratios (95% CI) for Nucleocapsid Reactivity	*P* Value	Multivariable Adjusted Risk Ratios (95% CI) for Nucleocapsid Reactivity	*P* Value
Age				
<50 y	1.0 (ref)	…	1.0 (ref)	…
50–64 y	0.95 (0.79–1.14)	.57	1.02 (0.84–1.23)	.85
65–79 y	0.95 (0.79–1.14)	.56	1.00 (0.82–1.23)	.97
≥80 y	1.34 (1.10–1.63)	<.01	1.29 (1.02–1.63)	.03
Sex				
Female	1.0 (ref)	…	1.0 (ref)	…
Male	1.02 (0.92–1.13)	.73	1.05 (0.94–1.18)	.35
Income				
<50k	1.0 (ref)	…	1.0 (ref)	…
50–100k	1.00 (0.88–1.14)	.99	1.06 (0.92–1.22)	.43
>100k	0.79 (0.66–0.93)	.01	0.85 (0.70–1.03)	.10
Race/ethnicity				
Non-Hispanic White	1.0 (ref)	…	1.0 (ref)	…
American Indian	1.20 (1.06–1.37)	.00	1.21 (1.02–1.44)	.03
Asian	1.27 (0.90–1.79)	.17	0.81 (0.62–1.05)	.12
Black	1.06 (0.93–1.21)	.38	1.16 (0.81–1.65)	.42
Hispanic/Latino	0.87 (0.65–1.16)	.34	1.15 (1.01–1.32)	.04
Education attainment				
College or beyond	1.0 (ref)	…	1.0 (ref)	…
Less than high school	1.24 (1.03–1.50)	.02	1.12 (0.92–1.38)	.27
High school	1.23 (1.08–1.39)	.00	1.04 (0.91–1.20)	.56
Some college	1.14 (0.99–1.30)	.06	1.10 (0.96–1.25)	.18
Smoking history				
Never	1.0 (ref)	…	1.0 (ref)	…
Former	1.21 (1.08–1.35)	.00	1.15 (1.03–1.29)	.01
Current	0.99 (0.84–1.18)	.95	1.01 (0.86–1.19)	.92
Body mass index				
<25 kg/m^2^	1.0 (ref)	…	1.0 (ref)	…
25–29.9 kg/m^2^	0.98 (0.84–1.13)	.75	1.00 (0.87–1.16)	.97
30–34.9 kg/m^2^	1.01 (0.87–1.19)	.85	0.94 (0.81–1.10)	.45
>35 kg/m^2^	1.11 (0.95–1.29)	.19	0.98 (0.84–1.15)	.84
Diabetes				
No	1.0 (ref)	…	1.0 (ref)	…
Yes	1.12 (1.00, 1.25)	0.06	0.99 (0.88, 1.12)	.92
Hypertension				
No	1.0 (ref)	…	1.0 (ref)	…
Yes	1.18 (1.06–1.31)	.00	1.04 (0.93–1.16)	.48
Cardiovascular disease				
No	1.0 (ref)	…	1.0 (ref)	…
Yes	1.10 (0.94–1.29)	.22	.99 (0.85–1.15)	.89
Chronic obstructive pulmonary disease				
No	1.0 (ref)	…	1.0 (ref)	…
Yes	1.14 (0.96–1.36)	.14	1.10 (0.93–1.31)	.26
Log-transformed anti-S1 MFI (per 1-unit increment)	1.21 (1.15–1.28)	<.0001	1.44 (1.35–1.53)	<.0001
COVID-19 infection severity				
Not hospitalized	1.0 (ref)	…	1.0 (ref)	…
Noncritical hospitalization	0.01 (0.88–1.16)	.85	0.94 (0.82–1.07)	.36
Critical hospitalization	0.27 (1.09–1.57)	<.01	1.11 (0.92–1.33)	.27
COVID-19 vaccine status				
Not vaccinated	1.0 (ref)	…	1.0 (ref)	…
Vaccinated after infection	0.74 (0.66–0.83)	<.0001	0.44 (0.38–0.50)	<.0001
Vaccinated before infection	0.76 (0.65–0.90)	<.01	0.36 (0.29–0.43)	<.0001
Time between infection and DBS collection				
120–179 d	1.0 (ref)	…	1.0 (ref)	…
0–29 d	0.54 (0.26–1.11)	.09	0.83 (0.46–1.48)	.52
30–89 d	0.91 (0.73–1.14)	.41	0.86 (0.69–1.07)	.17
90–119 d	1.12 (0.90–1.38)	.31	1.14 (0.92–1.43)	.23
180–364 d	0.83 (0.72–0.97)	.02	0.89 (0.76–1.04)	.16
>365 d	0.71 (0.60–0.84)	<.0001	0.72 (0.61–0.87)	<.001

C4R, Collaborative Cohort of Cohorts for COVID-19 Research; CI, confidence interval; DBS, dried blood spot; MFI, mean fluorescence intensity.

### Acute Illness Severity

**Figure 2.**
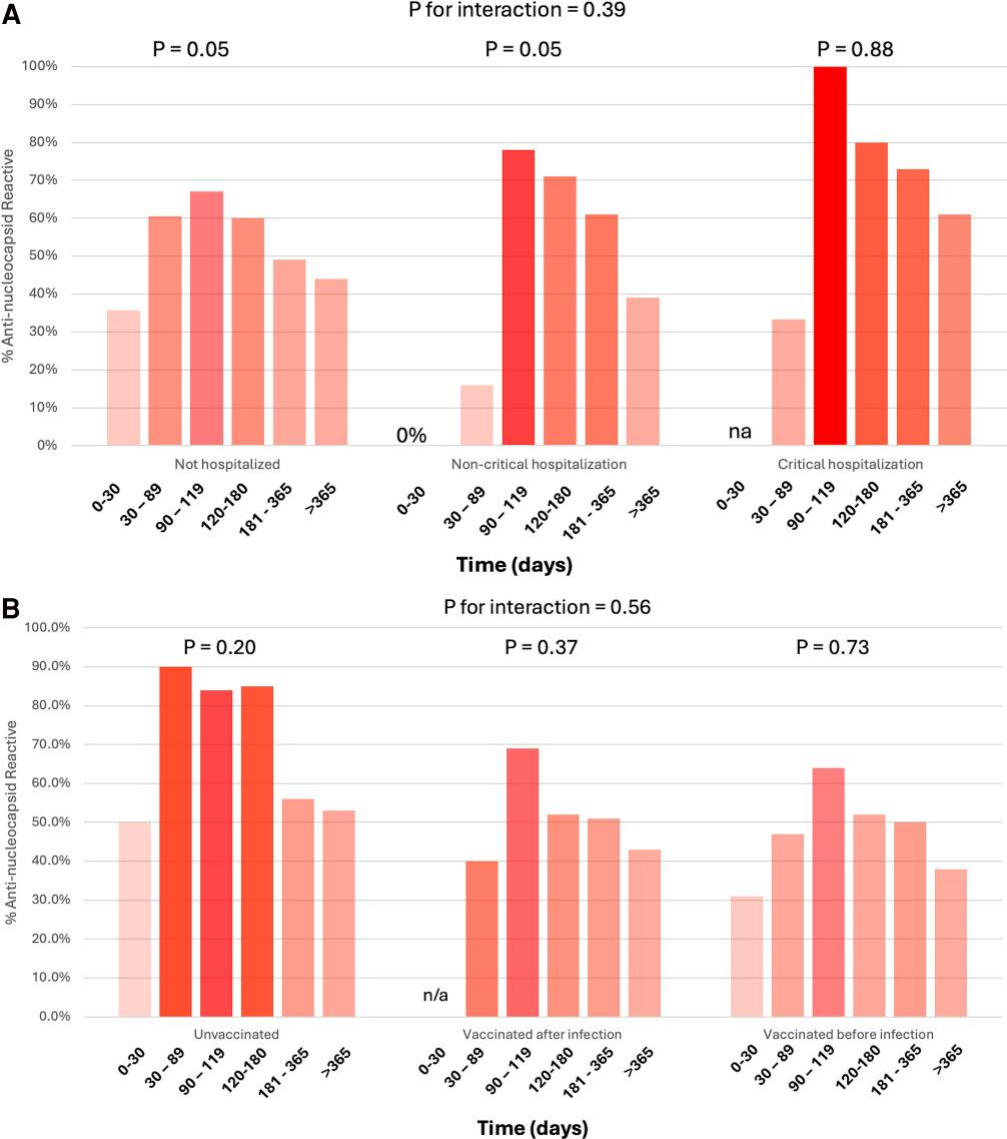
Proportion of individuals with anti-nucleocapsid antibody reactivity detected by time since infection according to severity of infection (*A*) and vaccination status (*B*) among n = 1419 C4R participants. *P* for interaction refers to the *P* value for a difference in reactivity over time by either hospitalization status or vaccination status. *P* refers to the *P* value for differences in reactivity over time within subgroups of hospitalization status or vaccination status.

Among hospitalized participants (vs not hospitalized), N-reactivity prevalence was lower in the first 3 months following infection but higher 3 months and beyond (*P* value for interaction between hospitalized COVID-19 and time since infection = 0.39, Figure 2*A*).

### Association Between Vaccination History and N-reactivity

Vaccination was associated with reduced serum IgG N-reactivity, whether or not individuals were vaccinated before (relative risk [RR] = 0.36; 95% confidence interval [CI], .29–.43) or after (RR = 0.44; 95% CI, .38–.50) infection. This observation remained after multivariable adjustment that included time since infection. Of note, time between infection and serosurvey differed among participants who were unvaccinated (275.9 ± 153.0 days), vaccinated after infection (377.0 ± 160.3 days), or vaccinated before infection (155.7 ± 129.8) (*P* < .0001, Supplementary Table 6 and Supplementary Figure 2). Although vaccination before or after infection was associated with a notably lower prevalence of reactivity overall, the lower prevalence was driven by results during the 1- to 6-month postinfection period, and prevalence estimates according to vaccination status were similar by 6 months after infection (Figure 2*B*, *P* for interaction = .56). There was not meaningful heterogeneity in correlates of nucleocapsid reactivity by vaccination status (Supplementary Table 7). Participant characteristics according to vaccination status are shown in Supplementary Table 6 and the distribution of time between infection and vaccination is shown in Supplementary Figure 3.

Vaccination manufacturer information was available among n = 995 participants, 94% of whom received an mRNA vaccination. With respect to vaccine manufacturer, compared to unvaccinated participants, nucleocapsid reactivity was reduced among individuals receiving either the Pfizer (RR = 0.76; 95% CI, .66–.86) or Moderna (RR = 0.76; 95% CI, .66–.87) vaccines; no differences in reactivity were observed between Pfizer and Moderna and too few participants were vaccinated with other non-mRNA vaccines to provide estimates.

## DISCUSSION

In a large, diverse, population-based cohort of US adults with prior SARS-CoV-2 infection, we observed that N reactivity peaked at 1–6 months before waning substantially, with just over half of participants infected ≥12 months before the serosurvey classified as nonreactive. Higher N reactivity was associated with several factors linked to a greater risk of severe COVID-19, including older age, former smoking, lower eduction and income, and lack of COVID-19 vaccination. In light of these results, the question of whether the time-dependent anti-N antibody response may offer information on the severity of SARS-CoV-2 viremia warrants further study.

The association of higher anti-N response with older age agrees with a previous study among a predominantly non-Hispanic White and generally healthy sample of >19 000 blood donors [9]. The present findings extend those results by including an older, more diverse cohort with a substantial prevalence of comorbidities and broader assessments of socioeconomic factors. Accordingly, we additionally observed that lower socioeconomic status (defined by education and income) was related to higher anti-N levels.

Prior studies has shown anti-N response among individuals with versus without hospitalization to be elevated [12, 15, 16, 26]. Although the small number of hospitalized infections in our sample limits statistical power, our findings support prior findings and suggest that hospitalization might be related to modestly elevated N-reactivity prevalence, which could be explained by greater viral exposure for prolonged periods. Interestingly, there was a trend suggesting that anti-N response was delayed in those hospitalized. However, this should be cautiously interpreted because there were few individuals with serologies shortly after hospitalization and the delayed response could also be explained by anti-COVID therapies administered in the hospital. Despite higher levels of reactivity following hospitalization, the pattern of antibody waning over time in this group was consistent with pattern observed in nonhospitalized participants. Our findings did not definitively show elevated anti-N levels in the full analysis (Figure 2*A*), possibly because most of the hospitalized participants had serology performed >6 months after infection. Future studies that measure anti-N trajectories longitudinally following infection will be important for confirming this finding.

The majority of factors associated with anti-N response following infection were inverse to our findings with respect to the anti-S1 response following vaccination. We previously reported that older age, male sex, current smoking, higher body mass index, non–mRNA-based vaccines, and certain comorbidities were associated with lower anti-S1 antibody levels after receiving COVID-19 vaccinations [2]. In contrast, several comorbidities were not related to anti-N response, whereas older age and former smoking were related to higher anti-N response and mRNA-based vaccines were related to lower anti-N response. We speculate that these seemingly opposing results can be reconciled as follows. Anti-S1 response to vaccination corresponds to the host immune response to a fixed S1 dose, whereas the anti-N response to a real-world viral exposure may correlate with the initial infectious dose and/or severity and duration of viremia which is exacerbated by risk factors such as older age and smoking.

Another important finding in our current study was the observation that vaccination was related to lower anti-N response regardless of timing vis-à-vis infection. This relationship was not observed in prior studies [7–9]. Knowledge about timing of infection provides C4R with a unique advantage when considering the role of vaccination in antibody response following infection because prior studies reporting on this association did not consider infection timing [8, 9]. Additionally, in 1 prior study showing no difference in anti-N response between vaccinated and unvaccinated health care workers, the time duration between vaccination and serological testing was very short (<35 days in all participants [8]). It is possible that infection shortly after vaccination still results in greater infection severity and notable viremia as vaccine-induced immunity might still be developing, thus resulting in a greater anti-N response. Pfizer vaccine trial results demonstrated that infection rates did not start to decrease until ∼2 weeks following the first vaccine dose [27], and prior serostudies report that seropositivity following vaccination is not present in the majority of participants until 12 days after vaccination [28].

The observation that vaccination, even after infection, is related to lower N-reactivity (compared to the unvaccinated) is somewhat counterintuitive as individuals unvaccinated at the time of infection may have higher viral loads and more severe infections, thus provoking a stronger antibody response. This pattern was most evident among those completing the serosurvey within 1–3 months of their infection, and as time since infection increased, reactivity prevalence estimates equalized across all groups.

There are several mechanisms by which vaccination after infection might influence antibody response. First, vaccination after infection could reduce viral persistence and chronic immune system provocation ongoing after recovery from the acute infection. Prior studies have found evidence for viral persistence for months following recovery from SARS-CoV-2 infection [29]. There is also evidence to suggest that postinfection vaccination could reduce signs and symptoms of post-acute sequelae of SARS-CoV-2, which is consistent with idea that vaccination can reduce viral persistence and dampen the sustained immune response to infection [30, 31]. Second, vaccination might redirect the immune system towards anti-S1 production and away from anti-N production suggesting a “zero-sum immunobiology.” A recent study in mice demonstrated that in stressed mice, SARS-CoV-2 vaccination resulted in increased antibody-binding affinity to the immunogen but decreased IgG levels and B-cell clonal expansion [32]. Third, being vaccinated after infection was possibly related to either the severity of infection and/or infection with the Omicron variant and given the attenuated virulence of Omicron, the immune response might have been attenuated as well. Finally, although confounding related to the differential time since infection could produce spurious findings, the distribution of time since infection was very similar between the unvaccinated and those vaccinated after infection and findings remained after adjustment for time since infection.

Beyond their potential biological and clinical applications, the present findings have important implications for future research studies and clinical practice. Most notably, that there are clear correlates of anti-N response raises the potential for differential information bias in studies of COVID-19. For example, the use of antibody results to assess prior infection in clinical practice, perhaps to rule in or out the potential for long-COVID, could lead to biased diagnostic patterns in which vaccinated individuals are less likely to receive a long-COVID diagnosis when compared to unvaccinated individuals. Additionally, although seroprevalence studies are known to underestimate prior infection estimates, these underestimates will be more pronounced among vaccinated, younger, and more affluent populations and those with less severe prior infections.

### Strengths and Limitations

C4R is a prospective, multiethnic, US general population-based sample that has comprehensive prepandemic phenotyping. Our data extend information on temporal trends because >40% of the cohort provided DBS >1 year following infection. Additionally, C4R data provide an integrated definition of prior infection using self-report and physician-adjudicated hospital records. The use of mailed DBS kits increased our response rates, particularly among groups who were less likely to leave the home for in-person study visits during early portions of the pandemic. The use of a semiquantitative assessment of anti-N IgG responses using validated methods is an important strength.

Some key limitations should be noted. First, information on neutralizing antibodies, other immunoglobulin isotypes (IgM, IgA), or measures of cellular immunity were not available. Second, a lack of repeated within person measures creates the potential for the observed time trends to be confounded by demographic and phenotypic factors. Third, the clinical significance of anti-nucleocapsid antibody response is yet to be determined and our high threshold for making a reactive call, while enhancing specificity, limits sensitivity as compared to other assays [17]. Fourth, misclassification of time-varying risk factors is possible. Clinical conditions and smoking status were measured several years before the serosurvey, which may conservatively bias our estimates. Infection history was defined primarily by self-report and could be subject to false-positive or false-negative classification, with uncertain influence on our estimates. Moreover, misclassification of infection due to recall bias could have been differential by levels of putative risk factors. We also were unable to confirm the absence of reinfections between the index infection and serological assays. Fifth, the high rate of overweight/obesity in this study sample might limit the generalizability of findings to populations with less overweight/obesity. Similarly, our sample does not adequately represent severely immunocompromised populations. Finally, our definition of anti-N IgG reactivity was internally derived and may not reflect reactivity thresholds used in other studies.

In conclusion, we observed modest levels of reactivity to an anti-N IgG antibody assay in a large, diverse population-based sample of US adults reporting history of SARS-CoV-2 infection. Older age, lower socioeconomic status, former smoking, increased anti-S1 response, hospitalization, and being unvaccinated were related to higher MFI levels and reactivity prevalence. Future research that can assess longitudinal within-person patterns will be necessary to better understand predictors of antibody response trajectories and their biological underpinnings, which may include differences in severity and duration of viremia. Studies that can assess serological response to a primary infection as a predictor of future risk for severe reinfection will also help to contextualize the present findings.

## Supplementary Material

ofaf123_Supplementary_Data

## Supplementary Data

Supplementary materials are available at *Open Forum Infectious Diseases* online. Consisting of data provided by the authors to benefit the reader, the posted materials are not copyedited and are the sole responsibility of the authors, so questions or comments should be addressed to the corresponding author.

## Note

***Author Contributions*.** We confirm that all coauthors have made appropriate contributions toward authorship and that they have approved the content of our manuscript. Author contributions are as follows: Concept and study design: All authors contributed to data collection, study design and conceptualization, interpretation of results, and reviewing the final version of the manuscript. Analysis of data: R.T.D., C.W., P.B., E.C.O. Writing the original manuscript draft: R.T.D., E.C.O.
