# Supplementary material for: Demographic and Clinical Factors Associated With SARS-CoV-2 Anti-Nucleocapsid Antibody Response Among Previously Infected US Adults: The C4R Study
Source: Open Forum Infect Dis. 2025 Mar 20;12(3):ofaf123. doi: 10.1093/ofid/ofaf123 (PMC11927777; doi:10.1093/ofid/ofaf123)
Supplement: ofaf123_Supplementary_Data [file ofaf123_supplementary_data.zip › SupplementalTable_1.pdf]

Supplemental Table 1. Baseline characteristics of C4R participants not included in the analysis due to missing serosurvey data

Characteristics	No. Participants, %
Total number of participants	29934
Reactivitiy	
Nmiss	2
Non-Reactive	1219 (56.6%)
Reactive	933 (43.4%)
Age	
Nmiss	145
Less than 50 years	3569 (12.0%)
50-64 years	8200 (27.5%)
65-79 years	10838 (36.4%)
80 years and greater	7182 (24.1%)
Sex	
Nmiss	46
Female	17428 (58.3%)
Male	12460 (41.7%)
Income	
Nmiss	29296
<50k	436 (68.3%)
50-100k	130 (20.4%)
>100k	72 (11.3%)
Race/ethnicity	
Nmiss	38
Non-Hispanic white	11363 (38.0%)
American Indian or Alaskan Native	973 (3.3%)
Asian	622 (2.1%)
Black	7639 (25.6%)
Hispanic	9279 (31.0%)
Others	20 (0.1%)
Education attainment	
Nmiss	827
College or beyond	10981 (37.7%)
Less than high school	5489 (18.9%)
High school	7196 (24.7%)
Some college	5441 (18.7%)
Smoking history	
Nmiss	167
Never	14907 (50.1%)
Former	10484 (35.2%)
Current	4376 (14.7%)
Body mass index	
Nmiss	439
<25 kg/m^2	6703 (22.7%)

25-29.9 kg/m^2	10833 (36.7%)
30-34.9 kg/m^2	6974 (23.6%)
>35 kg/m^2	4985 (16.9%)
Diabetes	
Nmiss	293
No	22961 (77.5%)
Yes	6680 (22.5%)
Hypertension	
Nmiss	145
No	11322 (38.0%)
Yes	18467 (62.0%)
Cardiovascular disease	
Nmiss	29043
No	679 (76.2%)
Yes	212 (23.8%)
Chronic Obstructive Pulmonary Disease	
Nmiss	6892
No	20715 (89.9%)
Yes	2327 (10.1%)
COVID-19 infection severity	
Nmiss	2139
Not hospitalized	2896 (10.4%)
Non-critical hospitalization	24065 (86.6%)
Critical hospitalization	728 (2.6%)
Vaccine status	106 (0.4%)
Nmiss	13848
Not vaccinated	4014 (25.0%)
Vaccinated after infection	1232 (7.7%)
Vaccinated before infection	10840 (67.4%)
