# Supplementary material for: Demographic and Clinical Factors Associated With SARS-CoV-2 Anti-Nucleocapsid Antibody Response Among Previously Infected US Adults: The C4R Study
Source: Open Forum Infect Dis. 2025 Mar 20;12(3):ofaf123. doi: 10.1093/ofid/ofaf123 (PMC11927777; doi:10.1093/ofid/ofaf123)
Supplement: ofaf123_Supplementary_Data [file ofaf123_supplementary_data.zip › SupplementalTable_2.pdf]

Supplemental Table 2. Characteristics of C4R participants in the complete case and imputed dataset

Characteristic	Complete Case(N=469)	Imputed Dataset(N=1419)
Reactivitiy		
Non-Reactive	222 (47.3%)	6960 (49.0%)
Reactive	247 (52.7%)	7230 (51.0%)
Age		
Less than 50 years	77 (16.4%)	1360 (9.6%)
50-64 years	156 (33.3%)	5560 (39.2%)
65-79 years	167 (35.6%)	5893 (41.5%)
80 years and greater	69 (14.7%)	1377 (9.7%)
Sex		
Female	301 (64.2%)	8678 (61.2%)
Male	168 (35.8%)	5512 (38.8%)
Income		
<50k	255 (54.4%)	6223 (43.9%)
50-100k	123 (26.2%)	4101 (28.9%)
>100k	91 (19.4%)	3866 (27.2%)
Race/ethnicity		
Non-Hispanic white	179 (38.2%)	8114 (57.2%)
American Indian or Alaskan Native	174 (37.1%)	2330 (16.4%)
Asian	8 (1.7%)	210 (1.5%)
Black	49 (10.4%)	2876 (20.3%)
Hispanic	59 (12.6%)	660 (4.7%)
Education attainment		
College or beyond	177 (37.7%)	5733 (40.4%)
Less than high school	49 (10.4%)	1087 (7.7%)
High school	125 (26.7%)	3697 (26.1%)
Some college	118 (25.2%)	3673 (25.9%)
Smoking history		
Never	230 (49.0%)	6351 (44.8%)
Former	159 (33.9%)	5765 (40.6%)
Current	80 (17.1%)	2074 (14.6%)
Body mass index		
<25 kg/m^2	85 (18.1%)	2968 (20.9%)
25-29.9 kg/m^2	167 (35.6%)	4786 (33.7%)
30-34.9 kg/m^2	112 (23.9%)	3350 (23.6%)
>35 kg/m^2	105 (22.4%)	3086 (21.7%)
Diabetes		
No	182 (38.8%)	6194 (43.7%)
Yes	287 (61.2%)	7996 (56.3%)
Hypertension		
No	340 (72.5%)	10821 (76.3%)
Yes	129 (27.5%)	3369 (23.7%)
Cardiovascular disease		
No	426 (90.8%)	12650 (89.1%)
Yes	43 (9.2%)	1540 (10.9%)
Chronic Obstructive Pulmonary Disease		
No	434 (92.5%)	12438 (87.7%)
Yes	35 (7.5%)	1752 (12.3%)
COVID-19 infection severity		
Not hospitalized	321 (68.4%)	11037 (77.8%)
Non-critical hospitalization	118 (25.2%)	2483 (17.5%)
Critical hospitalization	30 (6.4%)	670 (4.7%)
Vaccine status		
Not vaccinated	96 (20.5%)	2452 (17.3%)
Vaccinated after infection	317 (67.6%)	9530 (67.2%)
Vaccinated before infection	56 (11.9%)	2208 (15.6%)
Time between infection and DBS collection		
120-179 days	70 (14.9%)	1320 (9.3%)
0-29 days	7 (1.5%)	150 (1.1%)
30-89 days	28 (6.0%)	920 (6.5%)
90-119 days	13 (2.8%)	620 (4.4%)
180-364 days	179 (38.2%)	6080 (42.8%)
>365 days	172 (36.7%)	5100 (35.9%)

N=9 missing age, N=1 missing sex,N=883 missing income,N=1 missing race, N=58 missing education, N= 3 missing smoking status, N=29 missing bmi, N=10 missing hypertension, N = 17 missing diabetes, N=67 missing cvd, N=383 missing copd, N = 2 missing infection severity, N = 87 missing vaccination status
