# Supplementary material for: Demographic and Clinical Factors Associated With SARS-CoV-2 Anti-Nucleocapsid Antibody Response Among Previously Infected US Adults: The C4R Study
Source: Open Forum Infect Dis. 2025 Mar 20;12(3):ofaf123. doi: 10.1093/ofid/ofaf123 (PMC11927777; doi:10.1093/ofid/ofaf123)
Supplement: ofaf123_Supplementary_Data [file ofaf123_supplementary_data.zip › SupplementalTable_4.pdf]

Supplemental Table 4. Proportion of C4R participants with IgG antibody reactivity to nucleocapsid antigen (unadjusted results from Poisson models)

Group	0-30 days (n=15)	31-89 days (n=92)	90-119 days (n62)	120-180 days (n=132)	181-365 days (n=608)	>365 days (n=510)
Overall	33.33%	56.52%	69.35%	62.12%	51.81%	44.31%
Vaccine type						
Unvaccinated	50.00%	90.48%	84.21%	85.07%	56.21%	53.67%
Vaccinated after infection	n/a	40.00%	69.34%	52.22%	50.98%	43.32%
Vaccinated before infection	30.77%	46.97%	63.71%	51.87%	49.57%	37.69%
Age group						
Less than 50 years	50.00%	86.30%	71.43%	50.62%	31.10%	58.88%
50-64 years	66.67%	50.43%	75.00%	66.98%	47.07%	42.47%
65-79 years	12.50%	45.83%	46.67%	61.08%	53.66%	40.43%
80 years and greater	50.00%	71.43%	87.50%	59.82%	73.95%	58.90%
Sex						
Female	33.33%	61.82%	70.27%	63.33%	51.54%	42.13%
Male	33.33%	48.65%	68.00%	59.52%	52.19%	48.08%
Self-reported race or ethnicity						
Non-Hispanic White	20.00%	53.45%	69.77%	62.89%	50.77%	34.68%
African-American or Black	33.33%	54.55%	61.54%	70.59%	51.00%	49.44%
Hispanic	n/a	66.67%	n/a	37.50%	50.00%	35.48%
Asian	n/a	0.00%	0.00%	n/a	83.33%	42.86%
American Indian and Alaskan Native	100.00%	68.42%	100.00%	60.00%	53.66%	58.26%
Education attainment						
Less than high school	54.55%	61.54%	9.09%	71.43%	53.83%	58.20%
High school	69.70%	63.38%	77.05%	72.88%	55.56%	48.15%
Some college	31.82%	52.04%	69.01%	69.01%	52.11%	46.47%
College or beyond	1.96%	53.17%	66.90%	51.67%	48.83%	36.84%
Smoking status						
Never	42.86%	60.00%	71.33%	65.22%	46.22%	39.49%
Former	28.57%	56.41%	73.18%	61.19%	60.92%	48.63%
Current	0.00%	50.00%	50.00%	57.89%	42.42%	47.17%
Body mass index, kg/m^2						
<25 kg/m^2	9.09%	56.11%	68.57%	65.99%	49.78%	43.26%
25-29.9 kg/m^2	75.00%	48.67%	54.29%	58.87%	48.99%	44.32%
30-34.9 kg/m^2	24.49%	50.35%	79.88%	60.13%	50.34%	46.12%
>35 kg/m^2	7.69%	72.09%	88.68%	65.78%	60.86%	43.35%
Hypertension						
No	50.00%	56.10%	55.17%	55.17%	46.33%	40.39%
Yes	22.22%	56.86%	81.82%	67.57%	56.30%	47.10%
Diabetes						
No	39.66%	61.54%	69.17%	61.54%	48.20%	43.16%
Yes	11.76%	28.57%	70.30%	64.29%	62.57%	47.74%
Cardiovascular disease						
No	26.87%	57.09%	70.07%	62.08%	50.81%	44.11%
Yes	87.50%	52.29%	61.54%	62.37%	60.00%	46.17%
COPD						
No	33.56%	55.57%	70.84%	60.23%	50.55%	43.48%
Yes	n/a	61.87%	0.00%	83.33%	59.71%	49.92%
COVID-19 infection severity						
Not hospitalized	35.71%	60.46%	67.31%	60.00%	48.87%	44.44%
Non-critical hospitalization	0.00%	15.87%	77.78%	70.59%	61.39%	38.60%
Critical hospitalization	n/a	33.33%	100.00%	80.00%	72.73%	61.11%
