# Supplementary material for: Demographic and Clinical Factors Associated With SARS-CoV-2 Anti-Nucleocapsid Antibody Response Among Previously Infected US Adults: The C4R Study
Source: Open Forum Infect Dis. 2025 Mar 20;12(3):ofaf123. doi: 10.1093/ofid/ofaf123 (PMC11927777; doi:10.1093/ofid/ofaf123)
Supplement: ofaf123_Supplementary_Data [file ofaf123_supplementary_data.zip › SupplementalTable_5.pdf]

Supplemental Table 5. Correlates of anti-nucleocapsid antibody mean florescence intensity (MFI) levels after COVID-19 infection. Results from linear regression.

Clinical risk factor	Estimated mean percent difference in anti-N MFI (95% confidence interval)	P-value
Age		
Less than 50 years	0.0 (ref)	
50-64 years	-14.2% (-34.0% to 11.4%)	0.25
65-79 years	-5.3% (-28.4% to 25.2%)	0.70
80 years and greater	31.7% (-8.0% to 88.7%)	0.13
Sex		
Female	0.0 (ref)	
Male	15.0% (-1.1% to 33.8%)	0.07
Income		
<50k	0.0 (ref)	
50-100k	-3.6% (-22.7% to 20.2%)	0.75
>100k	-24.2% (-42.4% to -0.2%)	0.06
Race/ethnicity		
Non-Hispanic white	0.0 (ref)	
American Indian or Alaskan Native	57.6% (22.9% to 102.2%)	<0.001
Asian	-30.3% (-50.6% to -1.7%)	0.04
Black	-1.9% (-44.4% to 73.1%)	0.95
Hispanic	26.8% (4.7% to 53.6%)	0.02
Education attainment		
College or beyond	0.0 (ref)	
Less than high school	40.2% (3.8% to 89.3%)	0.03
High school	9.9% (-9.5% to 33.4%)	0.34
Some college	7.3% (-10.5% to 28.6%)	0.45
Smoking history		
Never	0.0 (ref)	
Former	23.6% (5.8% to 44.4%)	0.01
Current	17.6% (-4.7% to 45.1%)	0.13
Body mass index		
<25 kg/m^2	0.0 (ref)	
25-29.9 kg/m^2	-3.9% (-20.7% to 16.5%)	0.69
30-34.9 kg/m^2	-14.2% (-30.3% to 5.7%)	0.15
>35 kg/m^2	-3.2% (-22.4% to 20.8%)	0.77
Diabetes		
No	0.0 (ref)	
Yes	-3.7% (-18.6% to 14.1%)	0.67
Hypertension		
No	0.0 (ref)	
Yes	-2.7% (-16.3% to 13.2%)	0.73
Cardiovascular disease		
No	0.0 (ref)	
Yes	-3.7% (-23.2% to 20.8%)	0.75
Chronic obstructive pulmonary disease		

No	0.0 (ref)	
Yes	14.6% (-11.6% to 48.7%)	0.31
Log-transformed anti-S1 MFI (per 1-unit increment)	84.5% (73.1% to 96.7%)	< 0.0001
COVID-19 infection severity		
Not hospitalized	0.0 (ref)	
Non-critical hospitalization	-18.4% (-32.6% to -1.1%)	0.04
Critical hospitalization	6.6% (-23.0% to 47.5%)	0.70
COVID-19 vaccine status		
Not vaccinated	0.0 (ref)	
Vaccinated after infection	-80.9% (-84.8% to -76.1%)	< 0.0001
Vaccinated before infection	-87.1% (-90.4% to -82.7%)	< 0.0001
Time between infection and DBS collection		
120-179 days	0.0 (ref)	
0-29 days	-33.0% (-66.7% to 35.0%)	0.26
30-89 days	-5.5% (-33.9% to 35.0%)	0.75
90-119 days	51.3% (2.3% to 123.8%)	0.04
180-364 days	-26.5% (-43.1% to -5.0%)	0.02
>365 days	-42.1% (-56.1% to -23.8%)	< 0.0001
Batch 2 (Compare to Batch 1)	-24.4%(-36.3%, -10.2%)	<0.01
