# Supplementary material for: Demographic and Clinical Factors Associated With SARS-CoV-2 Anti-Nucleocapsid Antibody Response Among Previously Infected US Adults: The C4R Study
Source: Open Forum Infect Dis. 2025 Mar 20;12(3):ofaf123. doi: 10.1093/ofid/ofaf123 (PMC11927777; doi:10.1093/ofid/ofaf123)
Supplement: ofaf123_Supplementary_Data [file ofaf123_supplementary_data.zip › SupplementalTable_7.pdf]

Supplemental Table 7. Correlates of anti-nucleocapsid antibody reactivity after COVID-19 infection stratified by vaccination status.

Clinical risk factor	Vaccinated Before Infection Risk Ratio for anti-N Reactivity (95% confidence interval)	Vaccinated After Infection Risk Ratio for anti-N Reactivity (95% confidence interval)	Unvaccinated Risk Ratio for anti-N Reactivity (95% confidence interval)	P-value for difference by vaccination status
Age				0.85
Less than 50 years	1.0 (ref)	1.0 (ref)	1.0 (ref)	
50-64 years	0.90 (0.60, 1.35)	1.02 (0.77, 1.34)	1.09 (0.80, 1.50)	
65-79 years	0.67 (0.42, 1.07)	1.06 (0.79, 1.41)	1.14 (0.79, 1.63)	
80 years and greater	1.12 (0.66, 1.89)	1.28 (0.93, 1.75)	1.39 (0.89, 2.16)	
Sex				0.79
Female	1.0 (ref)	1.0 (ref)	1.0 (ref)	
Male	1.04 (0.77, 1.41)	1.07 (0.93, 1.23)	1.09 (0.87, 1.35)	
Income				0.93
<50k	1.0 (ref)	1.0 (ref)	1.0 (ref)	
50-100k	0.98 (0.58, 1.65)	1.11 (0.92, 1.34)	1.03 (0.79, 1.34)	
>100k	0.74 (0.45, 1.21)	0.91 (0.70, 1.18)	0.84 (0.55, 1.27)	
Race/ethnicity				0.42
Non-Hispanic white	1.0 (ref)	1.0 (ref)	1.0 (ref)	
American Indian or Alaskan Native	1.54 (1.03, 2.29)	1.44 (1.14, 1.82)	0.76 (0.54, 1.07)	
Asian	0.81 (0.27, 2.39)	1.33 (0.91, 1.96)	1.03 (0.66, 1.62)	
Black	1.23 (0.82, 1.83)	1.27 (1.06, 1.51)	0.98 (0.73, 1.31)	
Hispanic	0.51 (0.13, 2.05)	0.81 (0.56, 1.18)	1.00 (0.69, 1.45)	
Education attainment				0.99
College or beyond	1.0 (ref)	1.0 (ref)	1.0 (ref)	
Less than high school	1.11 (0.60, 2.05)	1.05 (0.79, 1.38)	1.21 (0.87, 1.70)	
High school	0.90 (0.60, 1.34)	1.01 (0.85, 1.21)	1.19 (0.92, 1.54)	
Some college	0.99 (0.68, 1.43)	1.08 (0.91, 1.29)	1.11 (0.87, 1.41)	
Smoking history				0.91
Never	1.0 (ref)	1.0 (ref)	1.0 (ref)	
Former	1.15 (0.85, 1.55)	1.21 (1.05, 1.40)	1.01 (0.81, 1.25)	
Current	1.05 (0.68, 1.61)	1.02 (0.82, 1.26)	0.98 (0.72, 1.33)	
Body mass index				0.60
<25 kg/m^2	1.0 (ref)	1.0 (ref)	1.0 (ref)	
25-29.9 kg/m^2	1.01 (0.66, 1.54)	0.98 (0.81, 1.17)	1.06 (0.81, 1.39)	
30-34.9 kg/m^2	1.17 (0.69, 1.99)	0.88 (0.73, 1.07)	1.06 (0.79, 1.40)	
>35 kg/m^2	1.19 (0.75, 1.88)	0.98 (0.79, 1.20)	0.95 (0.71, 1.26)	
Diabetes				0.37
No	1.0 (ref)	1.0 (ref)	1.0 (ref)	
Yes	0.82 (0.56, 1.21)	1.02 (0.88, 1.18)	1.00 (0.79, 1.26)	
Hypertension				0.53
No	1.0 (ref)	1.0 (ref)	1.0 (ref)	
Yes	1.04 (0.78, 1.39)	1.07 (0.92, 1.24)	0.90 (0.73, 1.12)	
Cardiovascular disease				0.98
No	1.0 (ref)	1.0 (ref)	1.0 (ref)	
Yes	0.98 (0.58, 1.64)	0.97 (0.79, 1.18)	1.00 (0.75, 1.32)	
Chronic obstructive pulmonary disease				0.61
No	1.0 (ref)	1.0 (ref)	1.0 (ref)	
Yes	0.84 (0.55, 1.28)	1.10 (0.89, 1.35)	1.33 (0.91, 1.92)	
Log-transformed anti-S1 MFI (per 1-unit increment)	2.85 (1.91, 4.24)	1.71 (1.51, 1.93)	1.25 (1.16, 1.34)	
COVID-19 infection severity				0.69
Not hospitalized	1.0 (ref)	1.0 (ref)	1.0 (ref)	
Non-critical hospitalization	1.10 (0.61, 2.00)	0.96 (0.82, 1.13)	0.72 (0.52, 0.98)	
Critical hospitalization	0.56 (0.09, 3.55)	1.14 (0.92, 1.40)	0.96 (0.70, 1.33)	
