# Supplementary material for: Demographic and Clinical Factors Associated With SARS-CoV-2 Anti-Nucleocapsid Antibody Response Among Previously Infected US Adults: The C4R Study
Source: Open Forum Infect Dis. 2025 Mar 20;12(3):ofaf123. doi: 10.1093/ofid/ofaf123 (PMC11927777; doi:10.1093/ofid/ofaf123)
Supplement: ofaf123_Supplementary_Data [file ofaf123_supplementary_data.zip › Supplemental_Table_3.pdf]

Supplemental Table 3. Baseline characteristics according to quartiles of anti-nucleocapsid antibody mean florescence intensity (MFI) levels

		Anti-N1 MFI Quartiles			
**Characteristic**	**Overall**	**Quartile 1**	**Quartile 2**	**Quartile 3**	**Quartile 4**
No. of participants	1419	354	355	355	355
Anti-S1 antibody MFI (log-transformed)	9.0 (1.3)	8.4 (1.6)	9.1 (1.1)	9.3 (1.0)	9.2 (1.1)
Anti-N antibody MFI (log-transformed)	7.3 (1.5)	5.3 (0.7)	6.8 (0.3)	7.9 (0.3)	9.1 (0.5)
% Reactive to Anti-S1	1393 (98.2%)	335 (94.6%)	351 (98.9%)	354 (99.7%)	353 (99.4%)
% Reactive to Anti-N	723 (51.0%)	2 (0.6%)	64 (18.0%)	307 (86.5%)	350 (98.6%)
Age					
Less than 50 years	132 (9.4%)	34 (9.6%)	28 (7.9%)	29 (8.3%)	41 (11.6%)
50-64 years	553 (39.2%)	161 (45.5%)	142 (40.1%)	134 (38.4%)	116 (32.9%)
65-79 years	588 (41.7%)	138 (39.0%)	158 (44.6%)	148 (42.4%)	144 (40.8%)
80 years and greater	137 (9.7%)	21 (5.9%)	26 (7.3%)	38 (10.9%)	52 (14.7%)
Female sex	867 (61.1%)	217 (61.3%)	214 (60.5%)	226 (63.7%)	210 (59.2%)
Income					
<50k	294 (54.9%)	59 (46.1%)	72 (55.8%)	77 (59.7%)	86 (57.3%)
50-100k	141 (26.3%)	36 (28.1%)	29 (22.5%)	36 (27.9%)	40 (26.7%)
>100k	101 (18.8%)	33 (25.8%)	28 (21.7%)	16 (12.4%)	24 (16.0%)
Self-reported race or ethnicity					
Non-Hispanic White	811 (57.2%)	216 (61.2%)	196 (55.2%)	191 (53.8%)	208 (58.6%)
African-American or Black	287 (20.2%)	68 (19.3%)	80 (22.5%)	69 (19.4%)	70 (19.7%)
Hispanic	66 (4.7%)	20 (5.7%)	20 (5.6%)	14 (3.9%)	12 (3.4%)
Asian	21 (1.5%)	3 (0.8%)	5 (1.4%)	8 (2.3%)	5 (1.4%)
American Indian and Alaskan Native	233 (16.4%)	46 (13.0%)	54 (15.2%)	73 (20.6%)	60 (16.9%)
Education attainment					
Less than high school	106 (7.8%)	18 (5.2%)	27 (7.9%)	31 (9.2%)	30 (8.9%)
High school	359 (26.4%)	72 (21.0%)	95 (27.8%)	93 (27.5%)	99 (29.3%)
Some college	354 (26.0%)	95 (27.7%)	81 (23.7%)	93 (27.5%)	85 (25.1%)
College or beyond	542 (39.8%)	158 (46.1%)	139 (40.6%)	121 (35.8%)	124 (36.7%)
Study cohort					
ARIC	70 (4.9%)	6 (1.7%)	13 (3.7%)	15 (4.2%)	36 (10.1%)
CARDIA	170 (12.0%)	46 (13.0%)	46 (13.0%)	35 (9.9%)	43 (12.1%)
COPDGene	187 (13.2%)	56 (15.8%)	49 (13.8%)	47 (13.2%)	35 (9.9%)
FHS	140 (9.9%)	42 (11.9%)	35 (9.9%)	24 (6.8%)	39 (11.0%)
JHS	26 (1.8%)	8 (2.3%)	4 (1.1%)	9 (2.5%)	5 (1.4%)
MASALA	12 (0.8%)	2 (0.6%)	3 (0.8%)	6 (1.7%)	1 (0.3%)
MESA	130 (9.2%)	43 (12.1%)	33 (9.3%)	26 (7.3%)	28 (7.9%)
PrePF	30 (2.1%)	7 (2.0%)	7 (2.0%)	9 (2.5%)	7 (2.0%)

REGARDS	345 (24.3%)	80 (22.6%)	93 (26.2%)	93 (26.2%)	79 (22.3%)
SARP	23 (1.6%)	2 (0.6%)	6 (1.7%)	7 (2.0%)	8 (2.3%)
SHS	231 (16.3%)	45 (12.7%)	54 (15.2%)	72 (20.3%)	60 (16.9%)
SPIROMICS	55 (3.9%)	17 (4.8%)	12 (3.4%)	12 (3.4%)	14 (3.9%)
Smoking status					
Never	633 (44.7%)	173 (48.9%)	161 (45.5%)	151 (42.8%)	148 (41.7%)
Former	576 (40.7%)	120 (33.9%)	148 (41.8%)	149 (42.2%)	159 (44.8%)
Current	207 (14.6%)	61 (17.2%)	45 (12.7%)	53 (15.0%)	48 (13.5%)
Body mass index, kg/m^2					
<25 kg/m^2	287 (20.6%)	69 (19.8%)	74 (21.4%)	73 (21.1%)	71 (20.3%)
25-29.9 kg/m^2	470 (33.8%)	119 (34.2%)	122 (35.3%)	119 (34.4%)	110 (31.4%)
30-34.9 kg/m^2	330 (23.7%)	81 (23.3%)	90 (26.0%)	79 (22.8%)	80 (22.9%)
>35 kg/m^2	303 (21.8%)	79 (22.7%)	60 (17.3%)	75 (21.7%)	89 (25.4%)
Hypertension	796 (56.5%)	184 (52.6%)	194 (55.0%)	211 (59.9%)	207 (58.5%)
Diabetes	334 (23.8%)	75 (21.4%)	84 (24.0%)	88 (25.1%)	87 (24.7%)
Cardiovascular disease	146 (10.8%)	30 (9.0%)	39 (11.4%)	39 (11.5%)	38 (11.2%)
COPD	118 (11.4%)	28 (10.6%)	27 (10.6%)	31 (12.4%)	32 (12.0%)
COVID-19 infection severity					
Not hospitalized	1102 (77.8%)	271 (77.0%)	283 (79.7%)	272 (76.6%)	276 (77.7%)
Non-critical hospitalization	248 (17.5%)	69 (19.6%)	56 (15.8%)	63 (17.7%)	60 (16.9%)
Critical hospitalization	67 (4.7%)	12 (3.4%)	16 (4.5%)	20 (5.6%)	19 (5.4%)
Vaccine status					
Not vaccinated	234 (17.6%)	41 (12.2%)	46 (13.9%)	52 (15.6%)	95 (28.7%)
Vaccinated after infection	891 (66.9%)	233 (69.1%)	237 (71.6%)	230 (69.1%)	191 (57.7%)
Vaccinated before infection	207 (15.5%)	63 (18.7%)	48 (14.5%)	51 (15.3%)	45 (13.6%)
Time since infection and DBS collection, months	12.3 (6.4)	11.3 (5.5)	10.5 (5.6)	9.8 (5.8)	11.0 (5.9)

Abbreviations: ARIC=Atherosclerosis Risk in Communities Study; CARDIA=Coronary Artery Risk Development in Young Adults, COPDGene=Genetic Epidemiology of Chronic Obstructive Pulmonary Disease, FHS=Framingham Heart Study; JHS=Jackson Heart Study; MASALA=Mediators of Atherosclerosis in South Asians Living in America; MESA=Multi-Ethnic Study of Atherosclerosis; MFI=mean fluorescence intensity; PrePF=Preclinical Pulmonary Fibrosis; REGARDS=Reasons for Geographic and Racial Differences in Stroke; SHS=Strong Heart Study

Data presented are from multiple imputation and percentages are rounded to nearest tenth decimal point.

Continuous variables presented as mean (standard deviation)

Categorical variables presented as number of participants (percentage)

Chronic kidney disease defined as estimated glomerular filtration rate below 45 $mL/min/1.73m^2$
