# Supplementary material for: Demographic and Clinical Factors Associated With SARS-CoV-2 Anti-Nucleocapsid Antibody Response Among Previously Infected US Adults: The C4R Study
Source: Open Forum Infect Dis. 2025 Mar 20;12(3):ofaf123. doi: 10.1093/ofid/ofaf123 (PMC11927777; doi:10.1093/ofid/ofaf123)
Supplement: ofaf123_Supplementary_Data [file ofaf123_supplementary_data.zip › Supplemental_Table_6.pdf]

Supplemental Table 6. Participant characteristics according to vaccination status vis-à-vis infection

Characteristic	Overall	Vaccination before Infection	Vaccination after Infection	Not Vaccinated
No. of participants	1332	207	891	234
Anti-S1 antibody MFI (log-transformed)	9.0 (1.3)	9.4 (1.1)	9.3 (0.9)	7.4 (1.6)
Anti-N antibody MFI (log-transformed)	7.3 (1.5)	7.0 (1.6)	7.2 (1.4)	7.8 (1.6)
% Reactive to Anti-S1	1306 (98.0%)	205 (99.0%)	886 (99.4%)	215 (91.9%)
% Reactive to Anti-N	671 (50.4%)	100 (48.3%)	422 (47.4%)	149 (63.7%)
Age				
Less than 50 years	124 (9.3%)	15 (7.3%)	70 (7.9%)	39 (16.7%)
50-64 years	522 (39.3%)	91 (44.2%)	342 (38.5%)	89 (38.2%)
65-79 years	558 (42.0%)	84 (40.8%)	387 (43.6%)	87 (37.3%)
80 years and greater	123 (9.3%)	16 (7.8%)	89 (10.0%)	18 (7.7%)
Female sex	808 (60.7%)	123 (59.4%)	545 (61.2%)	140 (59.8%)
Income				
<50k	268 (54.1%)	36 (56.2%)	168 (51.2%)	64 (62.1%)
50-100k	130 (26.3%)	13 (20.3%)	94 (28.7%)	23 (22.3%)
>100k	97 (19.6%)	15 (23.4%)	66 (20.1%)	16 (15.5%)
Self-reported race or ethnicity				
Non-Hispanic White	771 (57.9%)	121 (58.5%)	496 (55.7%)	154 (65.8%)
African-American or Black	270 (20.3%)	45 (21.7%)	200 (22.5%)	25 (10.7%)
Hispanic	62 (4.7%)	6 (2.9%)	43 (4.8%)	13 (5.6%)
Asian	19 (1.4%)	3 (1.4%)	15 (1.7%)	1 (0.4%)
American Indian and Alaskan Native	209 (15.7%)	32 (15.5%)	136 (15.3%)	41 (17.5%)
Education attainment				
Less than high school	97 (7.6%)	13 (6.6%)	67 (7.8%)	17 (7.6%)
High school	337 (26.4%)	50 (25.4%)	229 (26.8%)	58 (26.0%)
Some college	336 (26.3%)	52 (26.4%)	226 (26.4%)	58 (26.0%)
College or beyond	506 (39.7%)	82 (41.6%)	334 (39.0%)	90 (40.4%)
Study cohort				
ARIC	62 (4.7%)	1 (0.5%)	43 (4.8%)	18 (7.7%)
CARDIA	162 (12.2%)	32 (15.5%)	106 (11.9%)	24 (10.3%)
COPDGene	183 (13.7%)	58 (28.0%)	110 (12.3%)	15 (6.4%)
FHS	133 (10.0%)	9 (4.3%)	86 (9.7%)	38 (16.2%)
JHS	24 (1.8%)	8 (3.9%)	14 (1.6%)	2 (0.9%)
MASALA	11 (0.8%)	3 (1.4%)	7 (0.8%)	1 (0.4%)
MESA	120 (9.0%)	16 (7.7%)	88 (9.9%)	16 (6.8%)
PrePF	29 (2.2%)	5 (2.4%)	19 (2.1%)	5 (2.1%)
REGARDS	333 (25.0%)	25 (12.1%)	244 (27.4%)	64 (27.4%)

SARP	21 (1.6%)	5 (2.4%)	11 (1.2%)	5 (2.1%)
SHS	208 (15.6%)	32 (15.5%)	136 (15.3%)	40 (17.1%)
SPIROMICS	46 (3.5%)	13 (6.3%)	27 (3.0%)	6 (2.6%)
Smoking status				
Never	596 (44.8%)	77 (37.2%)	408 (45.8%)	111 (47.4%)
Former	544 (40.9%)	98 (47.3%)	358 (40.2%)	88 (37.6%)
Current	191 (14.4%)	32 (15.5%)	124 (13.9%)	35 (15.0%)
Body mass index, kg/m^2				
<25 kg/m^2	271 (20.8%)	44 (21.9%)	183 (20.9%)	44 (19.2%)
25-29.9 kg/m^2	445 (34.1%)	69 (34.3%)	292 (33.3%)	84 (36.7%)
30-34.9 kg/m^2	310 (23.7%)	43 (21.4%)	213 (24.3%)	54 (23.6%)
>35 kg/m^2	280 (21.4%)	45 (22.4%)	188 (21.5%)	47 (20.5%)
Hypertension	740 (56.0%)	109 (52.7%)	514 (58.1%)	117 (50.9%)
Diabetes	311 (23.6%)	39 (18.9%)	222 (25.1%)	50 (21.9%)
Cardiovascular disease	138 (10.8%)	16 (8.2%)	97 (11.3%)	25 (11.1%)
COPD	111 (11.6%)	23 (13.1%)	75 (12.0%)	13 (8.2%)
COVID-19 infection severity				
Not hospitalized	1054 (79.2%)	185 (89.8%)	681 (76.4%)	188 (80.7%)
Non-critical hospitalization	217 (16.3%)	18 (8.7%)	163 (18.3%)	36 (15.5%)
Critical hospitalization	59 (4.4%)	3 (1.5%)	47 (5.3%)	9 (3.9%)
Time since infection and DBS collection, months	9.2 (5.1)	12.6 (5.3)	5.2 (4.3)	10.8 (5.8)

n=211 participants excluded due to missing information on vaccination or infection dates
